# Randomised controlled trials of behavioural nudges delivered through text messages to increase influenza and COVID-19 vaccines among pregnant women (the EPIC study): study protocol

**DOI:** 10.1186/s13063-023-07485-9

**Published:** 2023-07-12

**Authors:** Prabha H. Andraweera, Bing Wang, Margie Danchin, Christopher Blyth, Ivo Vlaev, Jason Ong, Jodie Dodd, Jennifer Couper, Thomas R. Sullivan, Jonathan Karnon, Nicola Spurrier, Michael Cusack, Dylan Mordaunt, Dimi Simatos, Gus Dekker, Samantha Carlson, Jane Tuckerman, Nicholas Wood, Lisa Whop, Helen S. Marshall

**Affiliations:** 1 Vaccinology and Immunology Research Trials Unit, Women’s and Children’s Hospital, SA Health, Adelaide, South Australia Australia; 2 Robinson Research Institute, The University of Adelaide, Adelaide, South Australia Australia; 3 Adelaide Medical School, The University of Adelaide, Adelaide, South Australia Australia; 4 The Royal Children’s Hospital, Melbourne, Victoria Australia; 5 Department of Paediatrics, The University of Melbourne, Melbourne, Victoria Australia; 6 Perth Children’s Hospital, Perth, Western Australia Australia; 7 Department of Paediatrics, The University of Western Australia, Perth, Western Australia Australia; 8 School of Business, Warwick University, Warwick, UK; 9 School of Medicine, Monash University, Melbourne, Victoria Australia; 10 Women’s and Babies Division, Women’s and Children’s Hospital, Adelaide, South Australia Australia; 11 Division of Paediatrics, Women’s and Children’s Hospital, Adelaide, South Australia Australia; 12 South Australian Health and Medical Research Institute, Adelaide, South Australia Australia; 13 School of Public Health, The University of Adelaide, Adelaide, South Australia Australia; 14 Discipline of Public Health, Flinders University, Adelaide, South Australia Australia; 15 Discipline of Paediatrics, Flinders University, Adelaide, South Australia Australia; 16 SA Health, South Australian Government, Adelaide, South Australia Australia; 17 Discipline of Paediatrics, Lyell McEwin Hospital, Elizabeth Vale, South Australia Australia; 18 Discipline of Women’s Health, Lyell McEwin Hospital, Elizabeth Vale, South Australia Australia; 19 Murdoch Children’s Research Institute, Royal Children’s Hospital, Parkville, Victoria Australia; 20 Discipline of Paediatrics, University of Sydney, Sydney, New South Wales Australia; 21 Children’s Hospital Westmead, Sydney, New South Wales Australia; 22 Discipline of Public Health, Australian National University, Canberra, Australian Capital Territory Australia

**Keywords:** COVID-19, Influenza, Vaccine, Pregnancy, Randomised controlled trial, RCT

## Abstract

**Background:**

Influenza and COVID-19 infections during pregnancy may have serious adverse consequences for women as well as their infants. However, uptake of influenza and COVID-19 vaccines during pregnancy remains suboptimal. This study aims to assess the effectiveness of a multi-component nudge intervention to improve influenza and COVID-19 vaccine uptake among pregnant women.

**Methods:**

Pregnant women who receive antenatal care at five tertiary hospitals in South Australia, Western Australia and Victoria will be recruited to two separate randomised controlled trials (RCTs). Women will be eligible for the COVID-19 RCT is they have received two or less doses of a COVID-19 vaccine. Women will be eligible for the influenza RCT if they have not received the 2023 seasonal influenza vaccine. Vaccination status at all stages of the trial will be confirmed by the Australian Immunisation Register (AIR). Participants will be randomised (1:1) to standard care or intervention group (*n* = 1038 for each RCT). The nudge intervention in each RCT will comprise three SMS text message reminders with links to short educational videos from obstetricians, pregnant women and midwives and vaccine safety information. The primary outcome is at least one dose of a COVID-19 or influenza vaccine during pregnancy, as applicable. Logistic regression will compare the proportion vaccinated between groups. The effect of treatment will be described using odds ratio with a 95% CI.

**Discussion:**

Behavioural nudges that facilitate individual choices within a complex context have been successfully used in other disciplines to stir preferred behaviour towards better health choices. If our text-based nudges prove to be successful in improving influenza and COVID-19 vaccine uptake among pregnant women, they can easily be implemented at a national level.

**Trial registration:**

ClinicalTrials.gov Identifier NCT05613751. Registered on November 14, 2022.

**Supplementary Information:**

The online version contains supplementary material available at 10.1186/s13063-023-07485-9.

## Background

Pregnant women and their fetus or newborn infant are at increased risk of serious adverse consequences, hospitalisations and death from influenza and COVID-19 infections. A meta-analysis of 152 observational studies showed pregnant women are 2.4 times at higher risk of hospitalisation following influenza virus infection compared to the general population or non-pregnant women of reproductive age [1]. An Australian study demonstrated that pregnant or postpartum women with 2009 H1N1 influenza were at increased risk of admission to an intensive care unit compared with non-pregnant women of reproductive age (relative risk 7.4, 95% confidence interval (CI) 5.5 to 10.0). This risk was 13-fold greater (13.2, 95% CI 9.6 to 18.3) for women at 20 or more weeks’ gestation [2]. Of the pregnant women admitted to intensive care, two thirds required mechanical ventilation [2].

Pregnant women with COVID-19 infection are at increased risk of death, intensive care unit admission and major pregnancy complications compared to pregnant women who do not have COVID-19 infection in pregnancy [3–5]. In a living systematic review and meta-analysis of 435 studies of women with COVID-19 (pregnant or recently pregnant *n* = 293,152 and non-pregnant women of reproductive age *n* = 2,903,149), the odds are significantly higher for ICU admission (odds ratio (OR) 2.61 (95% CI 1.84 to 3.71) and invasive ventilation (2.41 (95% CI 2.13 to 2.71) among pregnant compared to non-pregnant women of reproductive age [6]. Pregnant women with COVID-19 also have increased odds of maternal death from any cause (OR 6.09, 95% CI 1.82 to 20.38) [6]. The odds of preterm delivery (OR 1.57, 95% CI 1.36 to 1.81), still birth (OR 1.81, 95% CI 1.38 to 2.37), caesarean section (OR 1.17, 95% CI 1.01 to 1.36) and admission of the newborn baby to neonatal ICU (OR 2.18, 95% CI 1.46 to 3.26) are also higher among pregnant women who have COVID-19 infection during pregnancy [6]. Pregnant women continue to be at increased risk of severe COVID-19 infection [7], with high BMI, advancing maternal age, non-white ethnicity, pre-existing comorbidities and pregnancy specific conditions including preeclampsia and gestational diabetes risk factors for severe disease [7].

Pregnant women are a group with special considerations for immunisation in an obstetric care hospital setting, regarding access to vaccine, provider recommendations (obstetricians and midwives who are not practicing immunisation providers) and timing of vaccination in relation to recommended gestation. Aboriginal and Torres Strait Islander pregnant women have been shown in multiple studies to have reduced uptake of recommended vaccines [8, 9]. Despite the adverse health consequences of both these infections in pregnancy, uptake of recommended influenza vaccine is only ~ 50% in pregnant women [10] and COVID-19 vaccine hesitancy among pregnant women is high [11]. A recent survey of 701 pregnant women showed that only 96 (13.7%) women accepted the COVID-19 vaccine, confirming that despite a high vaccine effectiveness, vaccine hesitancy among pregnant women is very high [12]. Safety issues around thrombotic events related to adenovector vaccine platforms highlight the rapidly changing immunisation landscape and heightened anxiety around vaccination in the population.

Improving uptake of recommended vaccines in people primarily attending hospitals requires a novel rather than a population approach. Interestingly, strategies aimed at educational interventions are less successful (or not successful at all) in improving vaccine uptake [13]. Over the past two decades, behavioural scientists have learnt how to design non-coercive “nudge” interventions to encourage positive behaviours in a range of contexts. Nudges are subtle changes in how choices are offered and provide an opportunity to “nudge” people to make better choices in a range of contexts. Nudges that facilitate individual choices within a complex context have been successfully used in other disciplines to stir preferred behaviour towards better health choices. Interestingly, strategies aimed at educational interventions are less successful (or not successful at all) in improving vaccine uptake [13].

A key concept of nudge design is actively designing the environment within which a choice is made to encourage better choices. The ideal choice environment is one that goes with the grain of individuals’ instincts or inherent cognitive and emotional biases to achieve better personal health goals. There are numerous such automatic cognitive biases that nudges (interventions) can trigger in order to produce desire changes in behaviours, such as, for example, our desire to avoid losses, imitate others, and maintain positive self-image. There are several straightforward nudges that health services could incorporate into vaccine programs such as framing vaccination as the default. Showing vaccination as the norm can activate social tendencies to join others. Making choices active and time-bound (for example, requiring people to accept or reject an appointment by a deadline) can boost acceptance rates. A previous study that nudged clinicians to accept or cancel orders for routine care such as vaccines or tests, found that this nudge alone increased influenza vaccination rates by 10% [14].

Nudges can be developed incorporating behavioural economic principles such as “foot in the door nudge” which asks the individual to perform a small request that has a high participation rate. This can be followed by a larger request, a “framing nudge” which relies on peoples’ tendency to make decisions differently depending on how the information is delivered and “social norm nudges” which highlight the common behaviours of one’s peers. The “nudge” process needs to be easy and low cost and if proven effective can be easily incorporated into standard patient care. For example, giving clinicians templated forms and patient lists of those under vaccinated could ensure completion of recommended vaccines and doses. A previous study found referrals for cardiac rehabilitation improved from 15 to 85% when a default clinician pathway was implemented [15].

This study aims to assess the effectiveness of a behavioural nudge intervention delivered through text messages on improving influenza and COVID-19 vaccine uptake among pregnant women using randomised controlled trials.

## Methods

### Nudge intervention

In August 2022, a nudgeathon was held to design the influenza and COVID-19 nudges. Twenty participants with diverse skills (pregnant women, obstetricians, midwives, hospital administrative personnel, behavioural scientists, psychologists and graphic designers) representing different organisations from South Australia, Western Australia and Victoria participated in the nudgeathon. The participants learnt about behavioural science and successful application of nudges in different fields. They were allocated into small groups, ensuring that each group comprised members with different skills. These individual groups were then asked to create a nudge for improving either COVID-19 or influenza vaccine uptake among pregnant women. The MINDSPACE framework which brings together numerous psychological influences on behaviour which are organised according to just nine fundamental principles (Messenger, Incentives, Norms, Defaults, Salience, Priming, Affect, Commitments, Ego) was used to identify potential nudges [16]. At the end of the nudgeathon, each group presented their ‘nudges’, and one nudge for each condition was selected by the team based on suitability for use in a hospital setting. The selected ‘nudge’ for both influenza and COVID-19 consists of three SMS text messages that are sent to pregnant women from the hospital 4 weeks apart (Messenger principle). The SMS messages will comprise a brief message that many pregnant women obtain the influenza/COVID-19 vaccine during pregnancy and a reminder for the woman to obtain the vaccine. Women have the option of responding to the message by (1) agreeing to obtain the vaccine, (2) stating that the participant has already received the vaccine or (3) requesting to opt out from receiving further reminders (first and second SMS messages). Each SMS message also provides a link to a video of the following: (1) a midwife stating the benefits of influenza/COVID-19 vaccination for pregnant women (first SMS); (2) a pregnant woman stating that she received the influenza/COVID-19 vaccine during pregnancy and that she and her new born baby are protected from serious adverse effects of influenza/COVID-19 infection (second SMS); (3) an obstetrician discussing the potential serious health consequences of influenza/COVID-19 infection in pregnancy (third SMS). In addition, the first SMS also provides a link to the current Australian Technical Advisory Group on Immunisation guidelines on influenza/COVID-19 vaccination in pregnancy. The first and second text messages also provide the option for women to agree to receive the vaccine or opt out from receiving further reminders.

### Primary objective

- ➣ To determine the difference in proportion of pregnant women in intervention versus standard care arm receiving one dose of the seasonal influenza/COVID-19 vaccine from the time of randomisation during pregnancy until delivery, as assessed using the Australian Immunisation Register (AIR).

### Secondary objectives

- ➣ To determine the difference in proportion of pregnant women in intervention versus standard care arm receiving one dose of the influenza/COVID-19 vaccine from the time of randomisation during pregnancy until one month after delivery, as assessed using the Australian Immunisation Register (AIR).
- ➣ To identify socio-demographic characteristics and medical risk factors associated with influenza/COVID-19 vaccination in pregnant women.
- ➣ To assess timeliness of influenza/ COVID-19 vaccine uptake among pregnant women during the study period by determining the proportion of pregnant women who receive the influenza/COVID-19 vaccine by month throughout the study period.
- ➣ Estimate the cost-effectiveness of proven interventions compared to standard care in hospital settings.

### Study design

This is a parallel-group randomised controlled trial (RCT, ClinicalTrials.gov Identifier: NCT05613751) that is designed to measure the impact of the nudge intervention on receipt of one dose of the influenza or COVID-19 vaccine in pregnant women who receive antenatal care at five metropolitan hospitals in South Australia (SA), Western Australia (WA) and Victoria. Pregnant women will be recruited at five hospitals across South Australia (Women’s and Children’s Hospital, Flinders Medical Centre and the Lyell McEwin Hospital), Victoria (Mercy Hospital for Women) and Western Australia (King Edward Memorial Hospital for Women). The three hospitals in SA represent the three South Australian health networks including Women’s and Children’s Health Network (WCHN), Southern Adelaide Local Health Network (SALHN) and Northern Adelaide Local Health Network (NALHN). Each of these hospitals reflect different socio-demographic areas and patients ensuring our research is generalizable. Influenza and COVID-19 vaccination status of pregnant women attending antenatal clinics of the hospitals will be ascertained by checking the Australian Immunisation Register (AIR). Those who have not received one dose of the influenza vaccine during pregnancy (in 2023) at the time of screening will be eligible for recruitment to the influenza vaccine RCT. Those who have received two or less doses of a COVID-19 vaccine will be considered to be not vaccinated or partially vaccinated in accordance with current COVID-19 vaccine recommendations and hence will be eligible for recruitment to the COVID-19 vaccine RCT. Exclusion criteria include women who have contraindications to influenza/COVID-19 vaccines and already randomised to one (influenza/COVID-19) RCT.

### Randomisation

Eligible pregnant women will be randomised to the intervention and standard care arms in a 1:1 ratio. The randomisation schedules will be prepared using R version 4.02 by an independent statistician who will not have a role in the conduct or analysis of the RCT. Allocations will be performed using randomly permuted blocks, stratified by hospital. Randomisation will be done by study coordinators on a password protected, web based, REDcap database held on the University of Adelaide server. The trial statistician will remain blinded.

### Study processes (Table 1)

**Table 1:** Schedule of enrolment, intervention and assessment

	**Study period**						
	**Enrolment**	**Allocation**	**Multi component nudge intervention**			**Primary Outcome**	**Secondary Outcome**
**Timepoint**	**1 week prior to antenatal clinic visit**		**1 week prior to antenatal clinic visit**	**4 weeks after M1**	**4 weeks after M2**	**Date of delivery**	**1 month after delivery**
**Enrolment**							
**Eligibility screen**	X						
**Allocation**		X					
**Interventions**							
**Message 1 (M1)**			X				
**Message 2 (M2)**				X			
**Message 3 (M3)**					X		
**Assessment**							
**Receipt of a dose of COVID-19/influenza vaccine**						X	X

Women attending antenatal clinics at the hospitals will be identified from Outpatients’ Department’s appointment lists. Research nurses will assess the influenza/COVID-19 vaccination status of women on AIR to screen for eligibility. Women will be ineligible if they have already received one dose of the influenza vaccine during their current pregnancy or three doses of a recommended COVID-19 vaccine or have no listed mobile phone number to receive a text message. Demographic and pregnancy outcome data will be obtained from hospital medical records. Women randomised to the intervention arm will receive a maximum of three text reminders using ‘Message Media’ software from the hospitals. The first SMS is sent approximately one week prior to a scheduled antenatal clinic visit. The second SMS message is sent four weeks after the first message to those who have not received a dose of the respective vaccine after the first SMS (confirmed on AIR) and not requested to opt out. The third SMS message is sent four weeks after the second message to those who have not received a dose of the respective vaccine after the second SMS (confirmed on AIR) and not requested to opt out. The AIR will be checked one month after delivery of the baby to assess whether women have received a dose of the influenza/COVID-19 vaccine after delivery. Pregnancy outcome data including date of delivery will be obtained from hospital medical records. All data will be stored on the REDCap database. All participants will be unblinded and the statisticians will be blinded. All data will be de-identified prior to presentation and publication.

### Study monitoring and surveillance

The nudge is a behavioural intervention and hence there are no risks associated with invasive procedures or medications. Any risk of psychosocial distress associated with receiving the SMS is negligible and the participants have the option of opting out from receiving the second and third SMS messages. A risk assessment and management plan has been developed from trial design to reporting stages. The study management committee comprising the chief investigator, site investigators, site coordinators and statistician will closely monitor the operational aspects of the study.

### Public involvement

Consumers including pregnant women and women of child bearing age participated in the nudgeathon to develop the nudge. The findings of the study will be communicated to key stakeholders and will be disseminated in peer-reviewed scientific journals and presented at national and international conferences.

### Ethics and dissemination

The protocol and all study material have been reviewed and approved by the Women’s and Children’s Health Network Human Research Ethics Committee (HREC/2022/00082) and research governance approval has been obtained from Women’s and Children’s Hospital, Flinders Medical Centre, Lyell McEwin Hospital, Mercy Hospital for Women and the King Edward Memorial Hospital for Women. A waiver of consent was approved for pregnant women to participate in this trial. The study will be conducted in accordance with the Declaration of Helsinki and the International Conference on Harmonization Guidelines on Good Clinical Practice. The trial will be conducted in compliance with the current version of the protocol. Any change to the protocol that affects scientific content, study design, participant willingness to participate will be considered an amendment and will be submitted to HREC for review and approval prior to implementation. After the completion of the study, the results will be presented at scientific forums and submitted for publication in peer-reviewed journals. The results will be disseminated regardless of the direction of effect. Authorship will be allocated according to the International Committees of Medical Journal Editors and the role of each author will be published in line with journal requirements. The study protocol was reported in accordance with the guidelines outlined in the SPIRIT Checklist for Trials [17].

### Sample size and statistical analysis plan

All comparisons will be undertaken assuming a standard superiority hypothesis testing framework. To detect an increase in the vaccination rate from ~ 50% in the control arm to 60% in the intervention arm, which is considered a clinically relevant impact, with 90% power (two-sided test with alpha = 0.05), a sample size of *n* = 519 per group (1038 total) is required. Should the vaccination rate in the control arm be higher than 50% or lower than 40%, this sample size will still provide at least 90% power to detect a 10% absolute increase in vaccination with the intervention. We will enrol at least 1038 pregnant women to each of the two RCTs across the participating hospitals.

Statistical analyses will be performed on an intention-to-treat basis according to a pre-specified statistical analysis plan. Baseline characteristics including age, socio-economic index based on post-code, ethnicity, parity, and other vaccines received during pregnancy will be reported for each group using percentages, means with standard deviations or medians with ranges as appropriate. The proportion vaccinated will be compared between randomised groups using logistic regression, with adjustment made for hospital. Treatment effects will be described as odds ratios with 95% confidence intervals. Patient characteristics associated with vaccination will be identified using multivariable logistic regression models.

Cost-effectiveness analysis of the nudge intervention will be compared to standard care from the healthcare payer perspective. The primary cost-effectiveness analysis will estimate the incremental cost per additional person vaccinated, in each of the two trials. Secondary analysis in study population will estimate the cost per quality-adjusted life year (QALY) averted on published literature. Vaccine uptake rates will be based on the study results. Implementation costs will be obtained from the study budget and costs related to research activities will be excluded. Estimated cost offsets to the health system associated with health service use (e.g. hospitalisations and emergency visits) will be obtained from hospital records and calculated using cost weights for Australian Refined Diagnosis Related Groups (AR-DRGs). A societal perspective sensitivity analysis will be undertaken using parent-reported out-of-pocket costs and productivity losses related to influenza illness for pregnant women and medically at-risk children. Out-of-pocket costs and indirect costs will be determined based on literature review. The economic evaluation will follow standard reporting guidelines in the CHEERS statement [18].

## Discussion

This study will assess the effectiveness of a multi-component nudge intervention on improving influenza and COVID-19 vaccine uptake among pregnant women.

A study of 20,000 pregnant women over 6 years in the USA, Australia Canada and Israel showed that there was a 40% reduction in hospitalisations from influenza among vaccinated individuals [19]. Influenza vaccination in pregnant women also reduces the risk of transmission to infants within the first few months of life [20]. However, influenza vaccine uptake among pregnant women in most countries still remains low. A recent systematic review identified (1) concerns about safety and risks to mother and child, (2) general low risk perception of becoming ill from influenza, (3) doubts about vaccine effectiveness, (4) lack of knowledge about the topic and (5) health care workers not providing adequate information about vaccination as some of the major reasons for the low influenza vaccine uptake among pregnant women [21]. Therefore, interventions that address these barriers are essential to improve influenza vaccine uptake among pregnant women.

Until mid-2021, there was insufficient data to recommend the routine use of COVID-19 vaccine for pregnant women. Due to the risk of severe outcomes from COVID-19 infection in pregnancy being significantly higher for pregnant women and their unborn babies, in June 2021, the Royal Australian and New Zealand College of Obstetricians and Gynaecologists and the Australian Technical Advisory Group on Immunisation advised that pregnant women should be offered the Pfizer Comirnaty messenger RNA vaccine at any stage of pregnancy [22, 23]. Despite strong evidence on mRNA COVID-19 vaccine safety in pregnancy [23], a recent systematic review and meta-analyses of 375 studies that included data from 25,147 participants reported that COVID-19 vaccine acceptance among pregnant women was only 49% [24]. A progressive decrease in vaccine acceptance was shown by income level (47% in high-income countries, 48% in middle-income countries and 61% in low-income countries) [24].

A previous RCT of a nudge intervention that included text message reminders (*n* = 93,354 participants) demonstrated that the first reminder increased appointment and COVID-19 vaccination rates within the healthcare system by 6.07 and 3.57% points and that the second reminder increased those by 1.65 and 1.06 points respectively [25]. Our team has recently shown a 47% relative increase in uptake of influenza vaccine in medically at-risk children using a simple SMS nudge co-designed with paediatricians [26]. If our multicomponent nudges prove to be successful in improving influenza and COVID-19 vaccine uptake among pregnant women, they can easily be implemented at a national level.

### Trial status

Current protocol version 2.0 (05–01-2023). Recruitment commencement date: 26 October 2022. Recruitment is expected to be complete by 31 December 2023.

## Supplementary Information

**Additional file 1.** SPIRIT Checklist for Trials.

## Data Availability

The data that support the findings of this study may be available from the corresponding author, HM, upon reasonable request.
